# Supplementary material for: Earlier Bedtime and Effective Coping Skills Predict a Return to Low-Risk of Depression in Young Adults during the COVID-19 Pandemic
Source: Int J Environ Res Public Health. 2022 Aug 18;19(16):10300. doi: 10.3390/ijerph191610300 (PMC9408272; doi:10.3390/ijerph191610300)
Supplement: Supplementary file 1 [file ijerph-19-10300-s001.zip › ijerph-1832689-supplementary.pdf]

Supplement to "Earlier Bedtime and Effective Coping Skills Predict a Return to Low-Risk of Depression in Young Adults during the COVID-19 Pandemic"

Qingyu Zhao, Kevin Wang, Orsolya Kiss (joined first authors), Dilara Yuksel, Massimiliano de Zambotti, Duncan B. Clark, David B. Goldston, Kate B. Nooner, Sandra A. Brown, Susan F. Tapert, Wesley K. Thompson, Bonnie J. Nagel, Adolf Pfefferbaum, Edith V. Sullivan, Kilian M. Pohl, Fiona C. Baker

Supplement Methods - Data Matching: Specific to the 119 participants that became high risk for depression by June 2020 (and were not before the pandemic), we created a matched dataset of those that did and did not return to low risk by 2021 using the maximum bipartite matching algorithm [1]. The method first constructed a bipartite graph such that the first set of nodes represented the 61 returning to low-risk participants while the second set of nodes presented the 58 persistent high-risk participants. An edge was connected between a returning and a persistent participant if they had the same CES-D-10 score in June 2020. The Ford-Fulkerson algorithm [2] then selected the maximum number of pairs matched with respect to the CES-D-10 score in June 2020. This process resulted in a matched dataset of 49 participants returning to low-risk and 49 persistent participants.

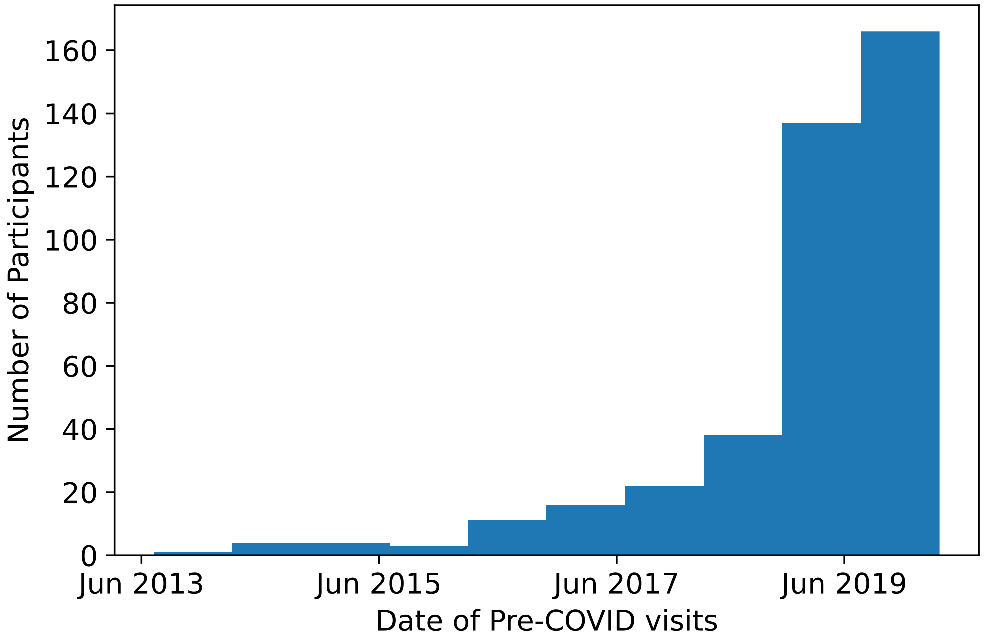

Supplementary Figure S1. Distribution of Pre-COVID visit date.

Supplementary Table S1. Results of the complete logistic regression model applied to the matched dataset of 49 participants returning to low risk in 2021 and the 49 persistent high-risk participants. Significant predictors are highlighted in bold (p<0.05).

	Estimate	*SE*	*tStat*	*pValue*
Intercept	0.48	0.887	0.541	0.589
Age	-0.315	0.478	-0.66	0.509
Sex	-0.557	0.357	-1.558	0.119
Age:sex	-0.73	0.423	-1.728	0.084
Study site				
Duke	0.296	1.235	0.24	0.811
OHSU	0.219	1.002	0.218	0.827
SRI	1.151	0.93	1.238	0.216
UPMC	1	1.014	0.986	0.324
Race				
Asian	0.331	1.167	0.284	0.777
African American	-0.034	1.3	-0.026	0.979
Others	-0.6	1.21	-0.496	0.62
Hispanic	**-3.238**	**1.359**	**-2.382**	**0.017**
Family socioeconomic status	0.02	0.328	0.062	0.95
Enrolled in education	-1.149	0.796	-1.444	0.149
Sleep behavior				
sleep quality	0.055	0.37	0.148	0.882
sleep bedtime	**1.172**	**0.463**	**2.533**	**0.011**
sleep duration	0.484	0.381	1.272	0.203
age:sleep quality	0.139	0.387	0.359	0.72
sex:sleep quality	-0.163	0.362	-0.451	0.652
age:sleep bedtime	-0.001	0.389	-0.002	0.999
sex:sleep bedtime	-0.173	0.405	-0.427	0.669
age:sleep duration	-0.595	0.406	-1.467	0.142
sex:sleep duration	-0.386	0.379	-1.02	0.308
Coping skills				
coping	0.231	0.355	0.651	0.515
age:coping	**-1.932**	**0.607**	**-3.183**	**0.001**
sex:coping	-0.098	0.357	-0.273	0.785
Alcohol use				
alcohol quantity	0.016	0.524	0.031	0.975
alcohol frequency	0.326	0.547	0.595	0.552
age:alcohol quantity	0.839	0.624	1.344	0.179
sex:alcohol quantity	0.436	0.434	1.004	0.315
age:alcohol frequency	-0.135	0.574	-0.236	0.813
sex:alcohol frequency	0.041	0.481	0.086	0.932

Supplementary Table S2. Results of the complete logistic regression model applied to 35 participants returning to CES-D≤7 and 58 persistent participants with CES-D-10≥10. Significant predictors are highlighted in bold (*p*<0.05).

	Estimate	*SE*	*tStat*	*pValue*
Intercept	-5.499	2.057	-2.674	0.007
age	0.417	0.685	0.609	0.543
sex	-0.148	0.445	-0.334	0.739
age:sex	-0.695	0.534	-1.3	0.194
study site				
Duke	2.124	1.581	1.343	0.179
OHSU	1.884	1.323	1.424	0.155
SRI	1.37	1.197	1.144	0.253
UPMC	1.613	1.449	1.114	0.265
race				
Asian	2.127	1.759	1.209	0.227
African American	0.027	2.133	0.013	0.99
Others	-0.784	1.334	-0.587	0.557
Hispanic	-1.405	1.629	-0.863	0.388
family socioeconomic status	-0.538	0.481	-1.118	0.263
enrolled in education	-0.108	1.118	-0.097	0.923
CES-D score in June 2020	**0.38**	**0.128**	**2.96**	**0.003**
Sleep behavior				
sleep quality	-0.249	0.441	-0.566	0.571
sleep bedtime	**1.264**	**0.596**	**2.122**	**0.034**
sleep duration	1.007	0.596	1.691	0.091
age:sleep quality	-0.033	0.413	-0.081	0.936
sex:sleep quality	-0.337	0.405	-0.832	0.405
age:sleep bedtime	0.485	0.592	0.819	0.413
sex:sleep bedtime	0.252	0.472	0.535	0.593
age:sleep duration	-0.255	0.526	-0.485	0.628
sex:sleep duration	0.293	0.522	0.561	0.575
Coping skills				
coping	-0.149	0.422	-0.353	0.724
age:coping	-0.967	0.516	-1.875	0.061
sex:coping	-0.152	0.427	-0.355	0.723
Alcohol use				
alcohol quantity	0.323	0.779	0.415	0.678
alcohol frequency	0.302	0.767	0.394	0.694
age:alcohol quantity	-0.049	0.825	-0.059	0.953
sex:alcohol quantity	0.317	0.639	0.496	0.62

age:alcohol frequency	0.956	0.766	1.249	0.212
sex:alcohol frequency	-0.253	0.703	-0.36	0.719

1. Zhao, Q., et al., *Association of Heavy Drinking With Deviant Fiber Tract Development in Frontal Brain Systems in Adolescents.* JAMA Psychiatry, 2021. **78**(4): p. 407-415.
2. Ford, L.R. and D.R. Fulkerson, *Maximal flow through a network. Canadian Journal of Mathematics*, 1956. **8**: p. 399-404.
